# Synthesis and *In Vitro* Antitumor Activity of Two Mixed-Ligand Oxovanadium(IV) Complexes of Schiff Base and Phenanthroline

**DOI:** 10.1155/2013/437134

**Published:** 2013-01-29

**Authors:** Yongli Zhang, Xiangsheng Wang, Wei Fang, Xiaoyan Cai, Fujiang Chu, Xiangwen Liao, Jiazheng Lu

**Affiliations:** ^1^Department of Biology, School of Basic Courses, Guangdong Pharmaceutical University, Guangzhou, Guangdong 510006, China; ^2^Guangdong Provincial Key Laboratory of Pharmaceutical Bioactive Substances, Guangzhou, Guangdong 510006, China; ^3^Chemistry Department, School of Pharmacy, Guangdong Pharmaceutical University, Guangzhou, Guangdong 510006, China

## Abstract

Two oxovanadium(IV) complexes of [VO(msatsc)(phen)], (**1**) (msatsc = methoxylsalicylaldehyde thiosemicarbazone, phen = phenanthroline) and its novel derivative [VO (4-chlorosatsc)(phen)], (**2**) (4-chlorosatsc = 4-chlorosalicylaldehyde thiosemicarbazone), have been synthesized and characterized by elemental analysis, IR, ES-MS, ^1^H NMR, and magnetic susceptibility measurements. Their antitumor effects on BEL-7402, HUH-7, and HepG2 cells were studied by MTT assay. The antitumor biological mechanism of these two complexes was studied in BEL-7402 cells by cell cycle analysis, Hoechst 33342 staining, Annexin V-FITC/PI assay, and detection of mitochondrial membrane potential (ΔΨm). The results showed that the growth of cancer cells was inhibited significantly, and complexes **1** and **2** mainly caused in BEL-7402 cells G0/G1 cell cycle arrest and induced apoptosis. Both **1** and **2** decreased significantly the ΔΨm, causing the depolarization of the mitochondrial membrane. Complex **2** showed greater antitumor efficiency than that of complex **1**.

## 1. Introduction

Schiff bases are an important class of ligands because such ligands and their transition metal complexes have a variety of applications including biological, clinical, and analytical applications [1]. The development of the field of bioinorganic chemistry has increased the interest in Schiff base complexes, because it has been recognized that N and S atoms play a key role in the coordination of transition metals at the active sites of many metallobiomolecules [2, 3]. The importance of metal ions in biological systems is well established. One of the most interesting features of metal-coordinated systems is the concerted spatial arrangement of the ligands around the metal ion [3–5]. 

Among the various transition metal ions used in pharmacological studies, Vanadium and its derivatives have been reported to display different biological effects including antitumor, antimicrobial, antihyperlipidemia, antihypertension, antiobesity, enhancement of oxygen affinity of hemoglobin and myoglobin, insulin-enhancing effects, and so on [6–8]. Vanadium complexes have also been explored for lowering of glucose levels [9–12], diuretic and natriuretic effects, antitumor activity against chemical carcinogenesis in animals and malignant cell lines (*in vitro*). Much effort has been done for vanadyl species coordinated to organic ligands on the research of their mimetic effects in hopes of developing vanadodrugs [13–15]. 

**Scheme 1:**
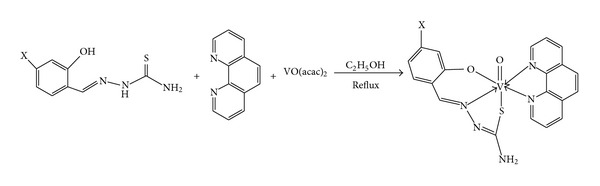
Synthesis of [VO(msatsc)(phen)] 1, X = –OCH_3_ and [VO(4-chlorosatsc)] 2, X = Cl.

Because V(IV) complexes have no charge, they are perceived to be candidates for easy bioabsorption. On the other hand, vanadyl(IV) complexes incorporating thiosemicarbazones have been studied extensively for their insulin-like effects which result in the inhibition of glycerol release and enhancement of glucose uptake by rat adipocytes and have been used in the treatment of tuberculosis [16–20]. In view of inquisitive response of oxovanadium(IV) in biology, we have reported that four oxidovanadium(IV) complexes present highly cytotoxic activities against Myeloma cell (Ag8.653) and Gliomas cell (U251) lines [21]. To continue our research in this project, in the present paper, an oxovanadium complex [VO(msatsc)(phen)] **1** (msatsc = methoxylsalicylaldehyde thiosemicarbazone, phen = phenanthroline) and its novel derivative [VO (4-chlorosatsc)(phen)] and (**2**) (4-chlorosatsc = 4-chlorosalicylaldehyde thiosemicarbazone) (Scheme 1) **2** have been synthesized and characterized. Their antitumor effects on BEL-7402 human liver (Bel7402), HUH-7, HepG2 cells were studied by MTT assay. The reported compounds may be an addition of new class of compounds as the metal-based drugs.

## 2. Experimental

### 2.1. Materials and Physical Measurements

VO(acac)_2_ (acac = acetylacetonate) and 1,10-phenanthroline (phen) were commercially available and used as received. DMSO, CHCl_3_ were purchased from Aldrich (USA). Other chemicals and reagents of analytical grade were obtained commercially without further purification unless specifically mentioned.

BEL-7402, HUH-7, and HepG2 cell lines were purchased from The Cell Bank of Type Culture Collection of Chinese Academy of Sciences (Shanghai, China). RPMI 1640 medium was purchased from Hyclone (Logan, USA), Trypsin, fetal calf serum and Annexin V-FITC/PI apoptosis detection Kit were purchased from GIBCO Company (USA). Hoechst 33342 staining solution was purchased from Beyotime Institute of Biotechnology (China). MTT and Rhodamine123 were purchased from Sigma Company (USA).

Microanalysis (C, H, and N) was carried out with a Perkin-Elmer 240Q elemental analyzer. Electrospray mass spectra (ES-MS) were recorded on a LCQ system (Finnigan MAT, USA) using methanol as mobile phase. ^1^H NMR spectra were recorded on a Varian-500 spectrometer. All chemical shifts are given relative to tetramethylsilane (TMS). Infrared spectra were recorded on a Bomen FTIR model MB102 instrument using KBr pellets. UV-Vis spectra were recorded on a Schimadzu UV-3101 PC spectrophotometer at room temperature. Emission spectra were recorded on a Perkin-Elmer Lambda 55 spectrofluorophotometer. Magnetic susceptibility measurements were recorded on a MPMSXL-7 (Quantum Design, USA), at room temperature. Solutions of compounds were freshly prepared 2 h prior to biochemical evaluation. 

Cell viability assay was carried out with a microplate reader (Model 680 Microplat Reader, BIO-RAD, USA). Cell cycle analysis, Annexin V-FITC/PI assay of apoptotic cells and detection of mitochondrial membrane potential were recorded on a FACScan flow cytometer (BD FACSCaliburTM, USA). Fluorescence microscopy of Apoptosis assays was carried out with the fluorescence microscope (Olympus OX31, Olympus Corporation, Japan).

### 2.2. Synthesis and Characterization

Methoxylsalicylaldehyde thiosemicarbazone (msatsc) and 4-chlorosalicylaldehyde thiosemicarbazone (4-chlorosatsc) were prepared with a method similar to that described earlier [19–21]. An equimolar methanolic solution of desired thiosemicarbazide (0.0182 g, 10 mmol) and corresponding methoxylsalicylaldehyde (0.0248 g, 10 mmol) or 4-chlorosalicylaldehyde (0.0560 g, 10 mmol) was refluxed for 3 h and then the precipitates were filtered off, washed with methanol and dried under vacuum. The products were recrystallized in ethanol. Msatsc: White solid. Yield: 80%. Anal. Calcd. for C_9_H_11_N_3_O_2_S: C, 48.01; H, 4.89; N, 18.67; S, 14.22%; Found: C, 47.89; H, 4.69; N, 18.53; S, 14.11%. ^1^H NMR (500 MHz; DMSO-d_6_; *δ*, ppm; s, singlet; d, doublet; t, triplet; m, multiplet): 11.34 (s, 2H, –NH), 9.88 (s, 1H, H–C=N), 7.21 (t, 1H, *J* = 7.1 Hz, –ph), 6.86 (d, 1H, *J* = 7.7 Hz, –ph), 6.81 (d, 1H, *J* = 7.6 Hz, –ph), 6.79 (t, 1H, *J* = 7.6 Hz, –ph), 3.53 (d, 3H, *J* = 7.5 Hz, –OCH_3_). ES-MS (CH_3_OH): *m/z *226.0 ([M+H]^+^). UV *λ*_max⁡_, nm (*ε*, M^−1 ^cm^−1^) in DMSO: 340 (26310). 4-chlorosatsc: White solid. Yield: 83%. Anal. Calcd. for C_8_H_8_N_3_OSCl: C, 42.01; H, 3.49; N, 18.38; S, 14.00%; Found: C, 41.85; H, 3.24; N, 18.13; S, 13.92%. ^1^H NMR (500 MHz; DMSO-d_6_; *δ*, ppm): 11.54 (s, 2H, –NH), 9.97 (s, 1H, H–C=N), 8.16 (s, 1H, –ph), 7.75 (s, 1H, –ph) 6.86 (d, 1H, *J* = 7.7 Hz, –ph),. ES-MS (CH_3_OH): *m/z *230.5 (M^+^). UV *λ*_max⁡_, nm (*ε*, M^−1^ cm^−1^) in DMSO: 346 (18655). 

#### 2.2.1. [VO(msatsc)(phen)]

A mixture of methoxylsalicylaldehyde thiosemicarbazone (msatsc) (0.0844 g, 0.375 mmol) and 1,10-phennanthroline·H_2_O (phen·H_2_O) (0.0743 g, 0.375 mmol) in absolute alcohol (20 cm^3^) were heated at 72°C under argon for 30 min. After dissolution, VO(acac)_2_ (0.1000 g, 0.375 mmol) was added and the brown red precipitate was formed immediately. This suspension was kept stirring under reflux for about 3.5 h and then the coloured solid formed was filtered off from the hot solution, washed with mixed solvents of ethanol and ether for three times and dried under vacuum. Yield: 90%. Anal. Calcd for C_21_H_17_N_5_O_3_SV: C, 53.63; H, 3.62; N, 14.89; S, 6.81%. Found: C, 53.54; H, 3.56; N, 14.63; S, 6.64%. ^1^H NMR (500 MHz; DMSO-d_6_; *δ*, ppm): 9.11 (d, 2H, *J* = 8.9 Hz, –NH), 8.51 (t, 1H, *J* = 9.5 Hz, H–C=N), 7.97 (d, 6H, *J* = 7.5 Hz, –phen), 7.78 (d, 2H, *J* = 7.8 Hz, –ph), 7.22 (t, 1H, *J* = 3.9 Hz, –ph), 6.86 (d, 1H, *J* = 7.7 Hz, –ph), 6.82 (d, 1H, *J* = 7.7 Hz, –ph), 6.77 (t, 1H, *J* = 7.6 Hz, –ph), 3.65 (d, 3H, *J* = 7.5 Hz, –OCH_3_). ES-MS (CH_3_OH): *m/z *470.9 ([M+H]^+^). UV *λ*_max⁡_, nm (*ε*, M^−1^ cm^−1^) in DMSO: 265 (41770), 340 (15255), 383 (6525), 760 (20), 797 (20). Magnetic moment: *μ*_eff_: 1.70 BM. Molar conductance *Ω*_M_ (Ω^−1^ cm^2^ mol^−1^): 9.65.

#### 2.2.2. [VO (4-chlorosatsc)(phen)]

This complex was synthesized with the same method described for **1**. Yield: 89%. Anal. Calcd for C_20_H_14_N_5_O_2_ClSV: C, 50.59; H, 2.95; N, 14.76; S, 6.75% Found: C, 50.45; H, 2.68; N, 14.53 S, 6.44%. ^1^H NMR (500 MHz; DMSO-d_6_; *δ*, ppm): 9.11 (d, 2H, *J* = 8.8 Hz, –NH), 8.51 (d, 1H, *J* = 8.1 Hz, H–C=N), 8.01 (s, 6H, –phen), 7.79 (d, 2H, *J* = 7.8 Hz, –ph), 7.78 (d, 3H, *J* = 7.9 Hz, –ph). ES-MS (CH_3_OH): *m/z *475.5 (M^+^). UV *λ*_max⁡_, nm (*ε*, M^−1^ cm^−1^) in DMSO: 264 (41015), 347 (10975), 400 (7815), 786 (20). Magnetic moment: *μ*_eff_: 1.73 BM. Molar conductance *Ω*_M_ (Ω^−1^ cm^2^ mol^−1^): 10.56. 

### 2.3. *In Vitro* Antitumor Activity

#### 2.3.1. Cell Culture

The cells were routinely cultured in RPMI-1640 medium, supplemented with 10% fetal calf serum. The culture was maintained at 37°C with a gas mixture of 5% CO_2_/95% air. The medium was changed every two days and the cells were subcultured every three days.

#### 2.3.2. Cell Viability Assay

Cell viability was determined using the MTT assay. Briefly, the cells were collected and resuspended in RPMI1640 medium at 4 × 10^4^ cells/mL, 100 *μ*L aliquots were added to each well of 96-well flat-bottomed microtiter plates, followed by addition of 100 *μ*L of the complexes **1** and **2**. Three replicate wells were used for each data point in the experiments. After incubation for the indicated intervals, 20 *μ*L of MTT (5 mg/mL in PBS) solution was added to each well and plates were then incubated for 4 h at 37°C. The medium with MTT was removed from the wells. Intracellular formazan crystals were dissolved by adding 150 *μ*L of DMSO to each well, and the plates were shaken for 10 min. The absorbance was read at 490 nm with a microplate reader. Percentage of survival was calculated as a fraction of the negative control (medium only). The half-maximal inhibitory concentration (IC_50_) was obtained from the dose-response curve with an original 6.0 software. 

#### 2.3.3. Cell Cycle Analysis

Analysis of the cell cycle of control and treated cancer cells was determined. Using standard methods, the DNA of cells was stained with PI, and the proportion of non-apoptotic cells in different phases of the cell cycle was recorded. The cancer cells were treated with complexes **1** and **2**, harvested by centrifugation at 1000 ×g for 5 min, and then washed with ice-cold PBS. The collected cells were fixed overnight with cold 70% ethanol, and then stained with PI solution consisting of 50 *μ*g/mL PI, 10 *μ*g/mL RNase. After 10 min incubation at room temperature in the dark, fluorescence-activated cells were sorted in a FACScan flow cytometer using CellQuest 3.0.1 software. 

#### 2.3.4. Fluorescence Microscopy of Apoptosis Assays

This method was modified from a previous report [18, 22]. Briefly, after exposed to complexes **1** and **2** for 48 h, BEL-7402 cells were washed twice with PBS, then stained with 10 *μ*g/mL Hoechst 33342 staining solution at 37°C for 30 min according to the manufacturer's instructions. Finally, the cells were observed under the fluorescence microscope.

#### 2.3.5. Annexin V-FITC/PI Assay of Apoptotic Cells

BEL-7402 cells treated with complexes **1** and **2** for 48 h were determined by flow cytometry using a commercially available Annexin V-FITC/PI apoptosis detection Kit. After treatment, cells were harvested and washed twice in ice-cold PBS and resuspended in 500 *μ*L of binding buffer at 1–5 × 10^5^ cells/mL. The samples were incubated with 5 *μ*L of Annexin V-FITC and 5 *μ*L propidium iodide in the dark for 15 min at room temperature. Finally, samples were analyzed by flow cytometry and evaluated based on the percentage of cells for Annexin V positive.

#### 2.3.6. Detection of Mitochondrial Membrane Potential (ΔΨm)

In this study, ΔΨm was measured by using Rhodamine123, and 5-Fluorouracil (5-FU, 50 *μ*M) is the positive control. Treated with complexes **1** and **2** for 48 h, BEL-7402 cells were incubated with Rhodamine123 (2 *μ*M/mL) at 37°C for 30 min, and washed with PBS. The cell pellets were collected by centrifugation (1000 ×g, 5 min) and resuspended in 500 *μ*L PBS. Green fluorescence intensities of Rhodamine 123 in cells were analyzed by FL-1 channel of the flow cytometer.

### 2.4. Statistical Analysis

Data were expressed as the mean ± SD from these independent experiments. Statistic analysis was performed using the SPSS 13.0 for Windows. Comparisons between two groups were performed by unpaired *t*-test. Multiple comparisons between more than two groups were performed by one-way analysis of variance (ANOVA). Significance was accepted at *P* value lower than 0.05. 

## 3. Results and Discussion

### 3.1. Complex Characterization

Oxovanadium(IV) complexes of formulation [VO(msatsc)(phen)] 1 (msatsc = methoxylsalicylaldehyde thiosemicarbazone, phen = phenanthroline) and its novel derivative [VO(4-chlorosatsc)(phen)] (4-chlorosatsc = 4-chlorosalicylaldehyde thiosemicarbazone) 2 are prepared in high yield from a general synthetic procedure in which vanadylacetylacetonate is reacted with the ligands in ethanol (Scheme 1).

To study the binding mode of the ligand to vanadium in the new complexes, IR spectra of the free ligands were compared with the spectra of the vanadium complexes. Selected IR data for complexes and their metal free ligands were given in Table 1. For the ligands, characteristic stretching vibration bands appear at 859 cm^−1^ and/or 829 cm^−1^ corresponding to the C=S vibration. A strong band is observed in the free ligands at 1614 cm^−1^ or 1603 cm^−1^, characteristic of the imine (C=N) [19, 23–25]. In the spectra of new complexes, the band due to **ν**(C=N) showed a red shift to 1625 or 1624 cm^−1^, indicating coordination of the nitrogen to vanadium (Table 1) [26, 27]. Medium intensity band, at 3467 and 3443 cm^−1^ in the free ligands due to *ν*_(OH)_, was absent in the complexes, indicating deprotonation of the Schiff base prior to coordination [25, 27]. In addition, the complexes exhibit the characteristic **ν**(V=O) bands at 965 and 940 cm^−1^, and **ν**(V–O) bands at 624 and 598 cm^−1^ for complex **1** and **2, **respectively. 

The ^1^H NMR spectra of complexes **1** and **2** are in excellent agreement with the proposed structures. In the ^1^H NMR spectra of the two complexes, the chemical shifts of the phenolic hydroxy protons of 11.34 ppm and 11.54 ppm, and amino protons linking directly to the imine groups of 9.88 ppm and 9.97 ppm for complexes **1** and **2**, respectively, in comparison with the metal-free ligands are unobserved. These facts also affirm that the free ligands are coordinated to metal. 

**Figure 1:**
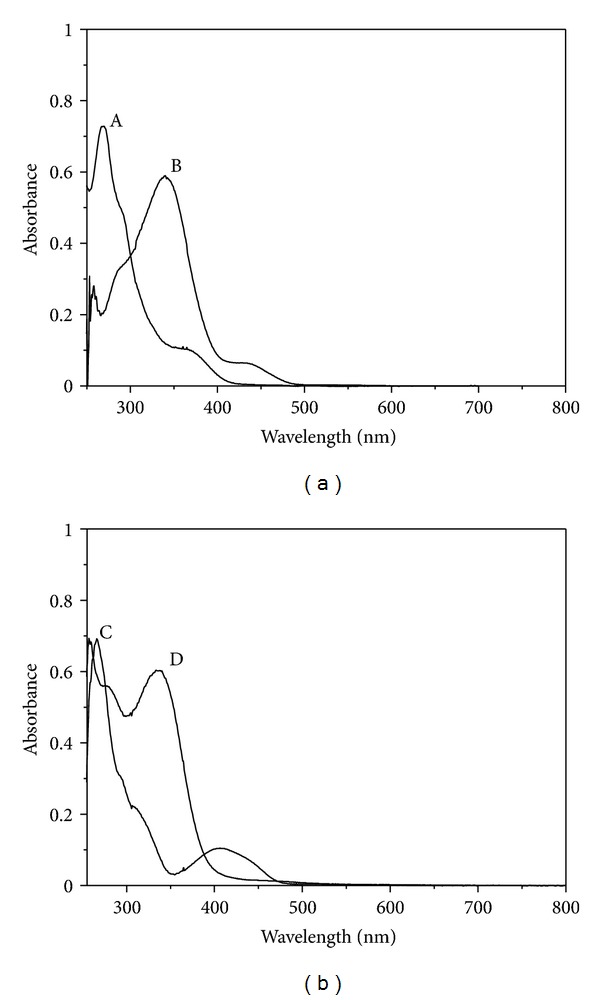
Absorption spectra of VO(msatsc)(phen) (A) and its ligand salsem (B) in (a) and VO(4-cholrobrsatsc)(*phen*) (C) and its ligand 4-cholrobrsatsc (D) in (b), respectively.

The electronic spectra of the two complexes and their ligands were shown in Figure 1. The complexes show an intense band at ca. 265 nm assignable to *π*-π* transitions of aromatic rings of phenanthroline [18–20, 28]. A medium band is observed near 400 nm, which is attributed to a ligand-to-metal charge-transfer transition (LMCT) as a charge transfer from a p-orbital on the lone-pair of ligands oxygen atoms to the empty d-orbital of the vanadium atom [27, 29]. The remaining bands appearing in the UV-region (320–350 nm) are assignable to the intraligand transitions of the Schiff base [18, 26–28]. Complexes of oxovanadium(IV) with coordination numbers 5 and 6 are usually square pyramidal/trigonal bipyramidal and distorted octahedral, respectively [19, 21, 29]. From the above obtained spectral data, it indicates that the Schiff bases bonded through the phenolate oxygen, imine nitrogen, and thiolate sulfur atoms leaving the thiomethyl as the pendant group. This implies that complexes **1** and **2** bear the central V (IV) atom in a square-pyramidal geometry [18, 29, 30]. 

The complexes are one-electron paramagnetic giving a magnetic moment value of ~1.70 BM at room temperature. These values of magnetic susceptibility also confirm that the vanadium complexes are in the V (IV) state, with d^1^ configuration [18, 19, 30, 31]. The molar conductance values of the two complexes in DMF indicated that these two oxidovanadium complexes show nonelectrolytic nature.

In addition, the assignments of the two complexes were made on the basis of elemental analyses and mass spectral data, confirming the proposed structures. The molecular ion peaks of complexes at *m*/*z* 441.1 and 475.6, for complexes **1** and **2**,respectively, were obtained by ESI-MS. 

### 3.2. *In Vitro* Antitumor Studies

#### 3.2.1. Antiproliferative Effect of the Complexes ****1**** and ****2**** on BEL-7402, HUH-7, and HepG2 Hepatoma Cells

**Figure 2:**
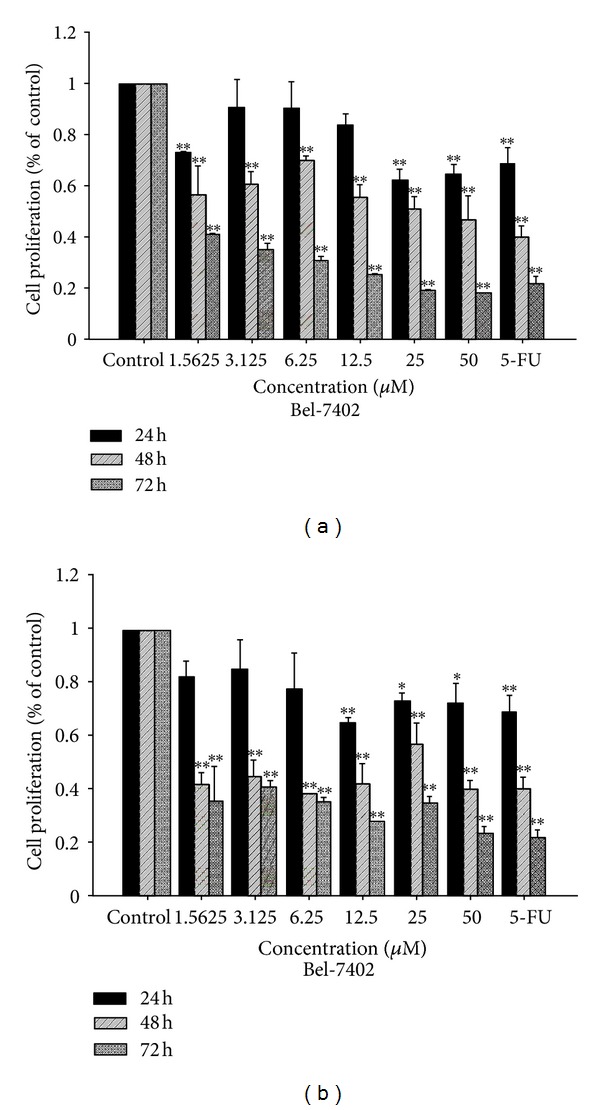
Antiproliferative activity of complexes **1** (a) and **2** (b) detected by MTT assay after 24, 48, and 72 h of treatment on BEL-7402 cells.

**Figure 3:**
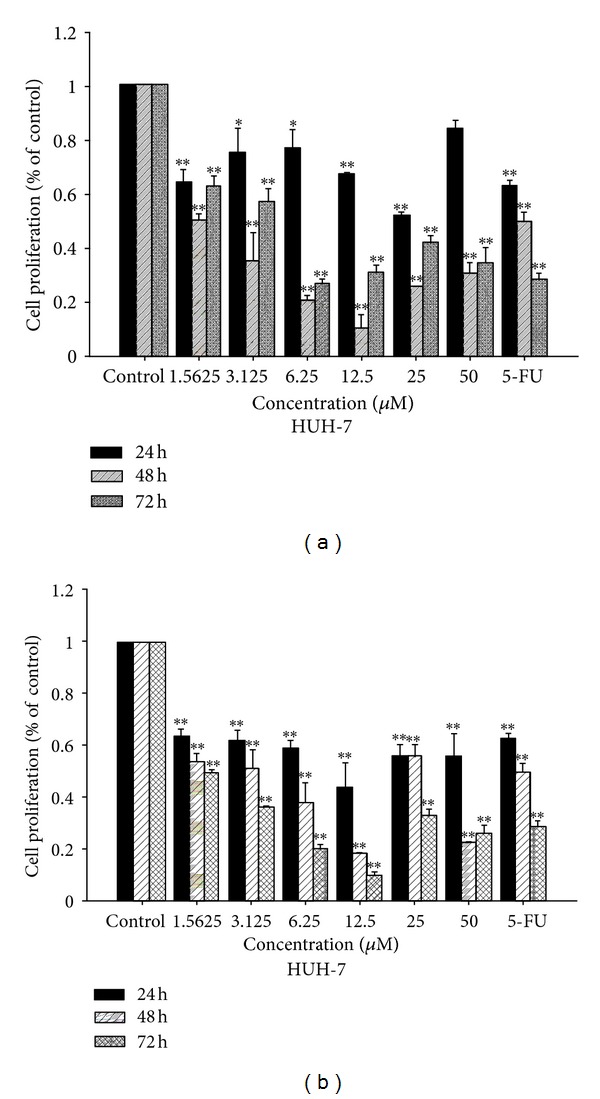
Antiproliferative activity of complexes **1** (a) and **2** (b) detected by MTT assay after 24, 48, and 72 h of treatment on HUH-7 cells.

**Figure 4:**
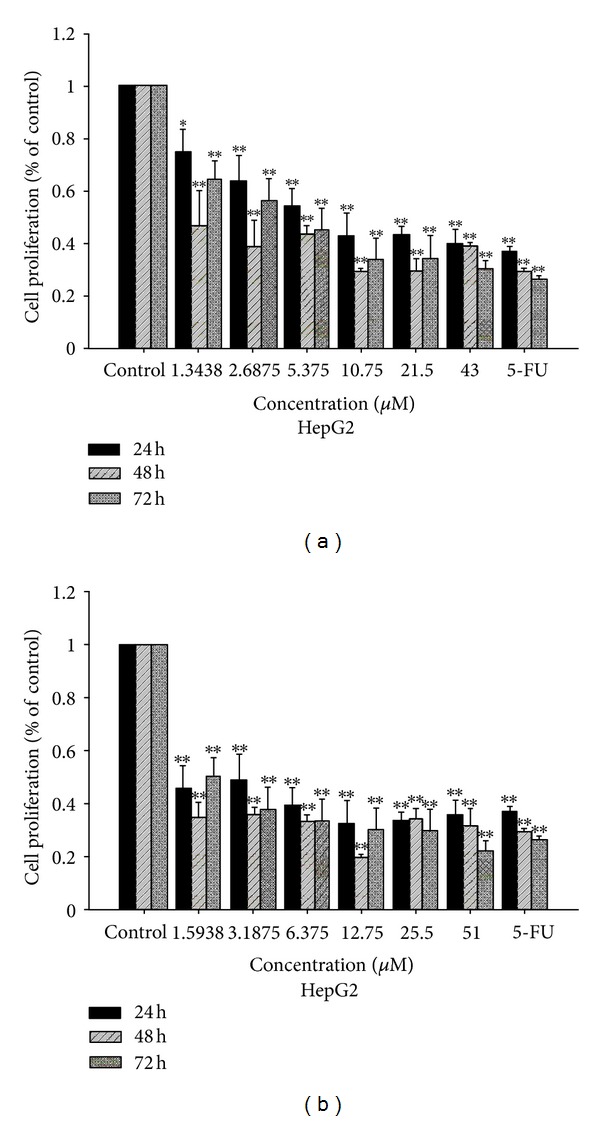
Antiproliferative activity of complexes **1** (a) and **2** (b) detected by MTT assay after 24, 48, and 72 h of treatment on HepG2 cells.

Complexes **1** and **2** were evaluated for their ability to inhibit the growth of BEL-7402, HUH-7, and HepG2 human hepatoma cell lines using MTT assay. The inhibition was expressed as cell viability relative to control without **1** and **2** treatments. In the present study, BEL-7402, HUH-7, and HepG2 human hepatoma cells were used which have been recently characterized as a suitable model for *in vitro* assessment of hepatoma toxicity [32, 33]. And 5-Fluorouracil (5-FU, 30 *μ*M) was used as a positive control, which has been used extensively as an efficient anticancer drug in clinical trials [32–34]. After treated for 24 h, 48 h, and 72 h on the selected three cell lines, the cells viability is showed in Figures 2, 3, and 4 and the IC_50_ values is summarized in Table 2.

As shown in Table 2 and Figures 2–4, the oxovanadium complexes exhibit broad inhibition on the three texted human cancer cell lines with the IC_50_ values ranging from 1.68 to 55.40 *μ*M, respectively. The results indicate that both of the oxidovanadium complexes **1** and **2** exhibit antiproliferative effect to human hepatoma cells BEL-7402, HUH-7, and HepG2 in a time and does-dependent manner with increasing the concentrations of **1** and **2**. The IC_50_ values of complex **2** on BEL-7402, HUH-7, and HepG2 cells after treated for 24 h, 48 h, and 72 h were less than that of complex **1 **(Figures 2–4, Table 2). It suggests that complex 2 possessed more potent inhibitory effect against the cancer cells. This difference may be attributed to the introduction of one chlorine on the 4 positions of the aromatic chromophore of salicylaldehyde thiosemicarbazone [18, 34, 35]. It implies that the electronic effect of salicylaldehyde thiosemicarbazone may be one of the factors in determining the anti-cancer activities. The results of the MTT-dye reduction assay unambiguously indicate that complexes **1** and **2 **exert potent cytotoxic/antiproliferative effect which in some cases is comparable to that of the referent cytotoxic drug 5-Fluorouracil. Among the cell lines under evaluation, the HUH-7 and HepG2 human hepatoma cells proved to be more sensitive to V(IV)-complexes treatment, actually the IC50 values in these cells were somewhat higher, but more or less comparable to that of 5-Fluorouracil.

#### 3.2.2. Cell Cycle Analysis

**Figure 5:**
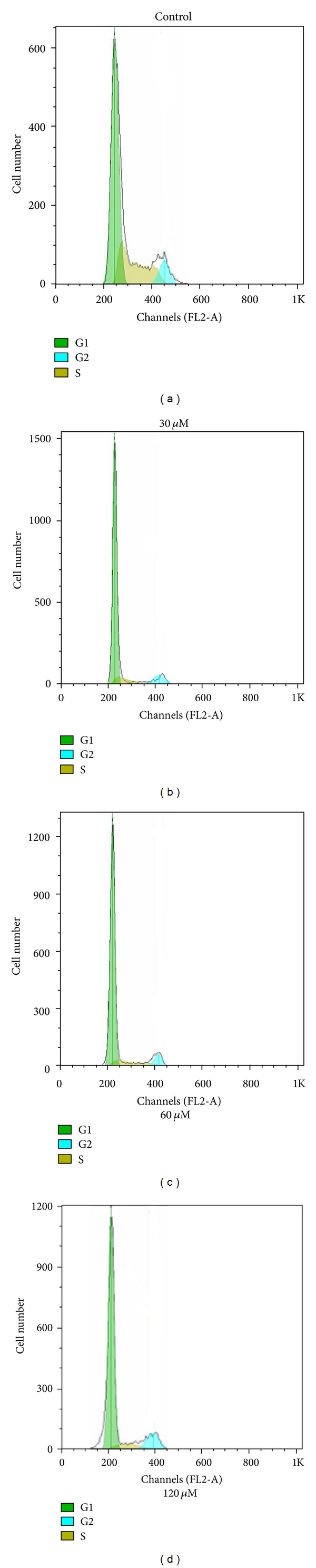
DNA content and cell cycle analysis of BEL-7402 cells after complex **1** treatment. BEL-7402 cells were cultured with either 0.1% DMSO (control), 30 *μ*M, 60 Mm, and 120 *μ*M of complex** 1** for 48 h. The percentage of nonapoptotic cells within each cell cycle was determined by flow cytometry.

**Figure 6:**
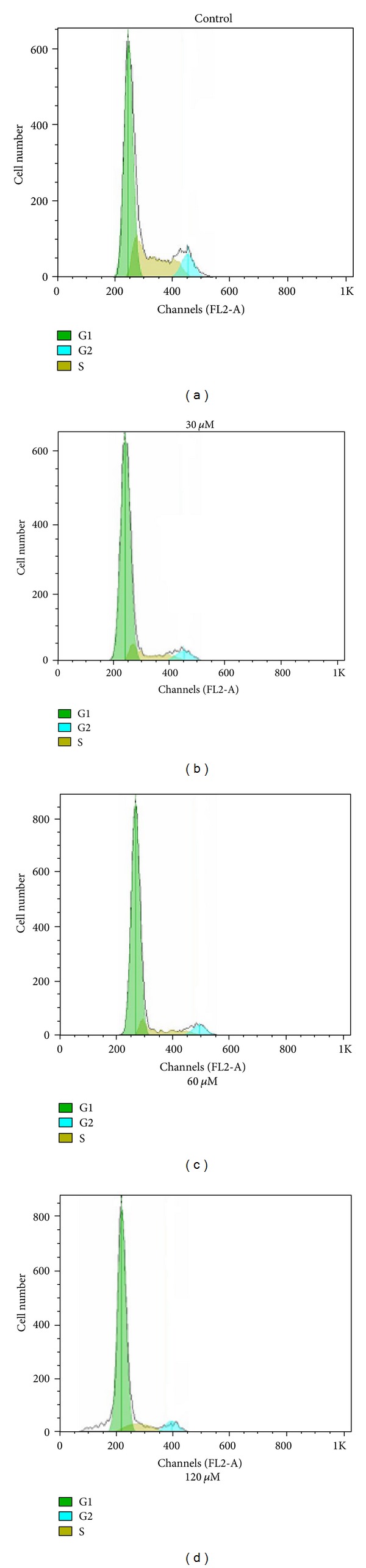
DNA content and cell cycle analysis of BEL-7402 cells after complex **2** treatment. BEL-7402 cells were cultured with either 0.1% DMSO (control), 30 *μ*M, 60 *μ*M, and 120 *μ*M of complex **2 **for 48 h. The percentage of nonapoptotic cells within each cell cycle was determined by flow cytometry.

In order to elucidate the mechanisms underlying the observed antiproliferative effect of complexes **1** and **2** on cancer cells, the cells cycle distribution was analyzed by flow cytometry with PI staining [17, 33–39]. The BEL-7402 cells were treated with 30 *μ*M, 60 *μ*M, and 120 *μ*M of **1** and **2** for 48 h, respectively. The results were shown in Figures 5 and 6. According to the results of Figures 5 and 6, it indicated that there were significantly increased rates of cells at G0/G1 phase and decreased the rates of cells at S and G2/M phase after BEL-7402 cells were exposed to **1 **and **2 **compared with untreated cells. 

As shown in Table 3, the oxovanadium complexes **1** and **2** inhibited growth of BEL-7402 cells by inducing a block in the G0/G1 phase of the cell cycle. The results in this work showed that there were significantly increased G0/G1 phase distribution and decreased G2/M phase distribution in a dose-dependent manner, indicating the induction of G0/G1-phase arrest by complexes **1** and **2**, and the arrested effect of **2** is stronger than that of **1**. The results also suggested that the two oxovanadium complexes induced proliferative suppression of BEL-7402 cells may be via the induction of apoptosis [34–38]. 

#### 3.2.3. Induction of Apoptosis as Evidenced by Hoechst 33342 Staining

Apoptosis is an important continuous process of destruction of undesirable cells during development or homeostasis in multicellular organisms. It is widely accepted that this process is characterized by distinct morphological changes including membrane blebbing, cell shrinkage, dissipation mitochondrial membrane potential (ΔΨm), chromatin condensation, and DNA fragmentationer [17, 18, 40, 41]. It has also been known that cancer was caused by the disruption of cellular homeostasis between cell death and cell proliferation [39, 42–44] and compounds which can induce apoptosis are considered to have potential as anticancer drugs [17, 18, 45].

**Figure 7:**
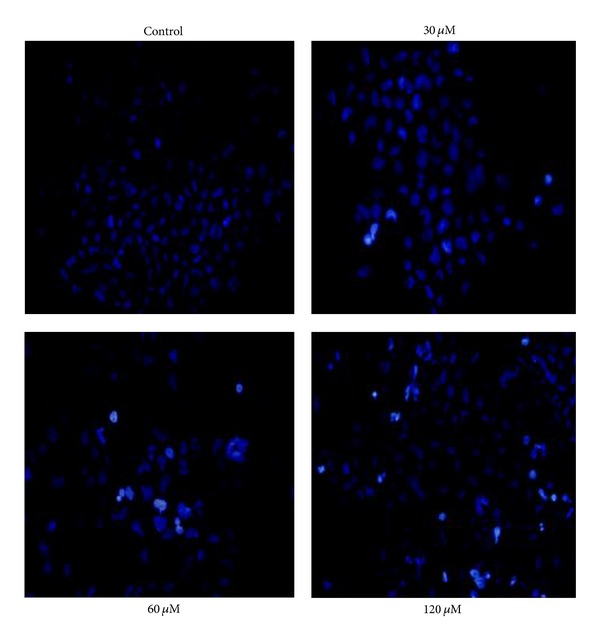
Effects of complex **1** on the morphology of Bel-7402 cells were assayed by Hoechst 33342 staining. After treatment with complex **1** for 48 h, apoptotic cells were detected by Hoechst 33342 staining and examined by fluorescence microscopy (original magnification 400x).

In an attempt to elucidate whether the G0/G1 phase arrest in the BEL-7402 cells induced by these complexes was associated with apoptosis, the occurrence of apoptosis by complexes **1** and **2** was identified with Hoechst 33342 staining. Figures 6 and 7 show representative Hoechst 33342 staining fluorescence photomicrographs of cultured BEL-7402 cells treated with or without **1** and** 2**, respectively. 

**Figure 8:**
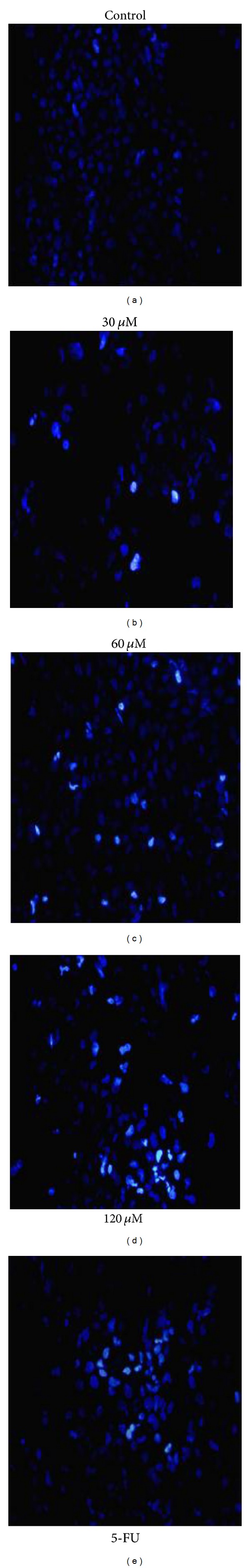
Effects of complex **2** on the morphology of Bel-7402 cells were assayed by Hoechst 33342 staining. After treatment with complex **2** for 48 h, apoptotic cells were detected by Hoechst 33342 staining and examined by fluorescence microscopy (original magnification 400x).

As shown in Figures 7 and 8, compared with the control, the BEL-7402 cells exhibited typical apoptotic features after treated with complexes **1** and **2** for 48 h including cellular morphological change, the apoptotic bodies, condensation of chromatin (brightly stained), and the apoptotic cells significantly increased in a dose-dependent manner with increasing the concentrations (30, 60, and 120 *μ*M, resp.) of **1** and **2**.

#### 3.2.4. Apoptosis Assessment by Annexin V-FITC/PI Assay

To further investigate the induced apoptosis effects of complexes **1 **and **2**, Annexin V-FITC/PI staining was performed to determine early, late apoptotic and necrotic cells, and 5-Fluorouracil (5-FU, 30 *μ*M) was used as the positive control. 

**Figure 9:**
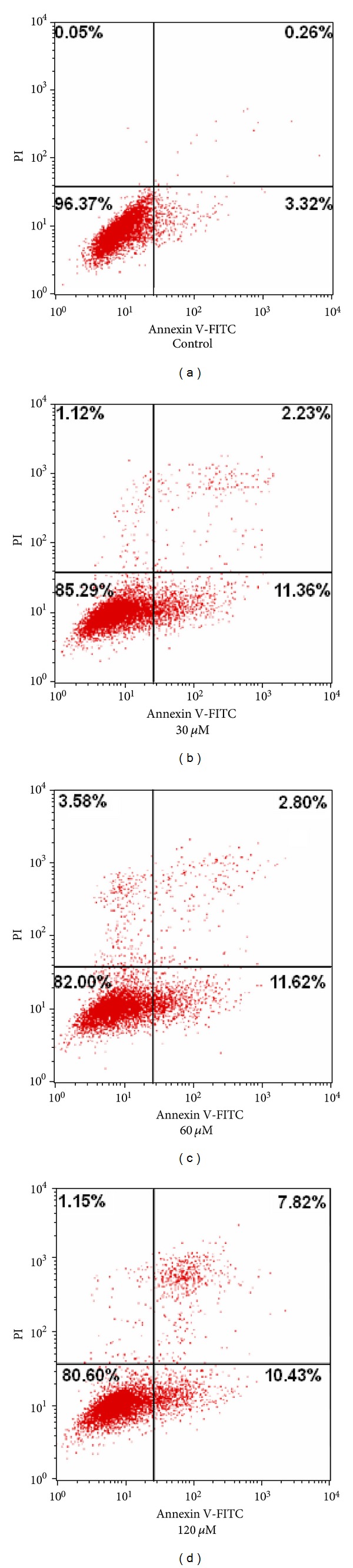
Distribution map of cell apoptosis. BEL-7402 cells were incubated with different concentrations of complex **1** (30, 60, and 120 *μ*M) for 48 h, subjected to Annexin V-FITC/PI staining, analyzed by flow cytometry.

**Figure 10:**
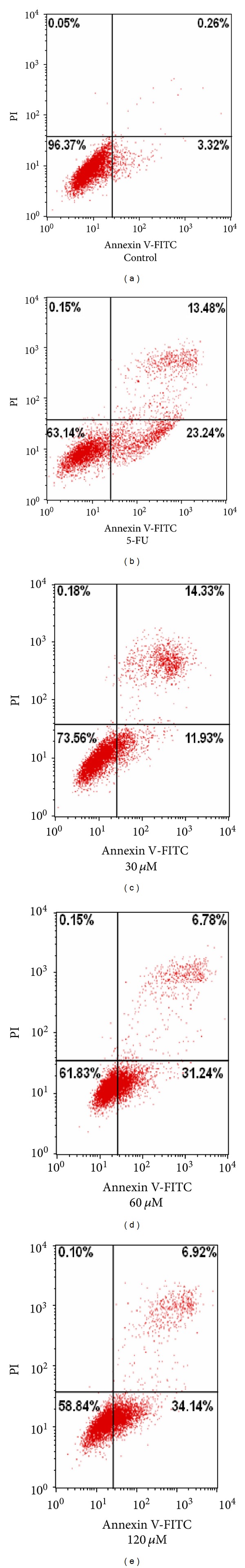
Distribution map of cell apoptosis. BEL-7402 cells were incubated with different concentrations of the complex **2** (30, 60, and 120 *μ*M) for 48 h, subjected to Annexin V-FITC/PI staining, and analyzed by flow cytometry.

It is known that Phosphatidylserine (PS) externalization is an early feature of apoptosis and can be detected by the binding of Annexin V to PS on the cell surface [40–45], and the apoptosis assessment method by Annexin V-FITC/PI assay is well recognized and accurate. As shown in Figures 9 and 10, with increasing amounts of complexes (30 *μ*M, 60 *μ*M, 120 *μ*M, resp.), the percentage of Annexin V-FITC/PI stained cells both the early and late apoptotic cells increases significantly. The results indicate that both complexes **1** and **2** induced proliferative suppression of BEL-7402 cells were via the induction of apoptosis, and the induced apoptosis effect of **2** is stronger than that of **1**. 

#### 3.2.5. Loss of Mitochondrial Membrane Potential (ΔΨm)

Disruption of the ΔΨm is one of the earliest intracellular events that occur following the induction of apoptosis [38–42], and it is also a hallmark for apoptosis. To evaluate the situation of mitochondria in **1** and **2** induced apoptosis, the ability of the two compounds to induce alterations in the mitochondrial membrane potential was investigated.

**Figure 11:**
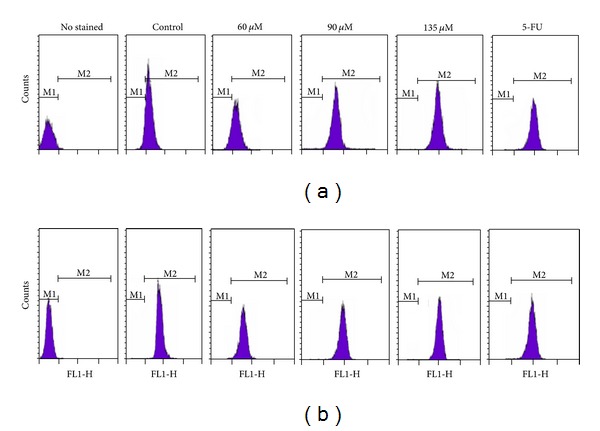
Distribution map of mitochondrial membrane potential with complex **1** (bottom) and complex **2** (top) for 48 h.

As shown in Figure 11 and Table 4 upon increasing the concentrations of complexes **1** and **2 **(60, 90, and 135 *μ*M), the mean value of green fluorescence intensity obtained from the flow cytometry on BEL-7402 cells increases steadily after treated for 48 h. It is indicated that a lot of Rhodamine 123 from mitochondria matrix was released to the cytoplasm in a dose dependent manner, complexes **1** and **2** can affect the mitochondrial function and causing the depolarization of the mitochondrial membrane, and lead to the ΔΨm value significantly decreased. The percentage relative to control of the complex **2** is stronger than that of the complex **1** (Table 4). These results imply that the induction of apoptosis by the complexes may be associated with the mitochondrial pathway [17, 22, 43–47]. However, further mechanism researches involved in the induction of apoptosis remain to be elucidated.

In summary, two oxovanadium(IV) complexes of [VO(satsc)(phen)] (**1**) (satsc = salicylaldehyde thiosemicarbazone, phen = phenanthroline) and [VO (4-chlorosatsc)(phen)] and (**2**) (4-chlorosatsc = 4-chlorosalicylaldehyde thiosemicarbazone) have been synthesized and characterized. Their antitumor effects on BEL-7402, HUH-7, and HepG2 cells were studied by MTT assay. *In vitro* experimental results show that both **1** and **2** possess significant antiproliferative effects. The results also showed that they can cause G0/G1 phase arrested of the cell cycle and decreased significantly the ΔΨm, causing the depolarization of the mitochondrial membrane. Both complexes exhibit significant induced apoptosis in BEL-7402 cells and displayed typical morphological apoptotic characteristics, and complex **2** showed greater antitumor efficiency than that of complex **1**. These results may provide further evidence to exploit the potential medicine compounds from the metal complexes.

## Figures and Tables

**Table 1:** Selected IR data for complexes and their corresponding ligands **ν**(cm^−1^).

Compound	*ν*(C=S)	*ν*(C–S)	*ν*(O–H)	*ν*(C=N)	*ν*(V=O)	*ν*(V–O)	*ν*(N–H)
VO(msatsc)(phen)	—	769	—	1625	965	624	3289
Msatsc	859	—	3467	1614	—	—	3356
VO(4-cholrobrsatsc) (phen)	—	767	—	1624	940	598	3284
4-Cholrobrsatsc	829	—	3443	1603	—	—	3319

**Table 2:** Comparison of IC_50_ values obtained from the MTT assay on BEL-7402, HUH-7, and HepG2 hepatoma cell lines after treated for 24 h, 48 h, and 72 h using the complexes **1 **and **2**.

Cell line	Subject	IC_50_ values obtained from the MTT assay (*μ*M)		
		24 h	48 h	72 h
BEL-7402	**1**	294.30 ± 95.74	30.80 ± 13.05	0.51 ± 0.21
	**2**	166.32 ± 20.48	17.02 ± 3.69	0.90 ± 0.13

HUH-7	**1**	69.91 ± 11.08	2.87 ± 0.23	3.12 ± 0.39
	**2**	79.16 ± 25.18	1.98 ± 0.72	0.28 ± 0.09

HepG2	**1**	12.21 ± 1.09	1.81 ± 0.38	5.02 ± 0.14
	**2**	4.32 ± 0.98	1.33 ± 0.37	1.71 ± 0.10

Data are the mean ± SD of at least three independent experiments. *P < 0.05; **P < 0.01. *P < 0.05 versus the control, the difference was significant. **P < 0.01 versus the control, the difference was markedly significant.

**Table 3:** The cell cycle analysis of the BEL-7402 cells induced by complexes **1 **and **2 **for 48 h.

Concentration (*μ*M)	The relative proportion of different phase in the cell cycle (%)			
		G0/G1	S	G2/M
Control		71.65 ± 1.81	14.28 ± 0.37	12.34 ± 1.78
	30	81.05 ± 0.23**	7.94 ± 0.81**	9.11 ± 0.57*
**1**	60	81.78 ± 0.42**	8.54 ± 0.40**	8.66 ± 1.03*
	120	81.96 ± 1.88**	8.18 ± 0.97**	8.58 ± 0.60*

	30	85.46 ± 2.58**	5.53 ± 0.23**	7.74 ± 1.82*
**2**	60	86.03 ± 1.23**	5.33 ± 0.20**	7.53 ± 1.21*
	120	86.00 ± 0.99**	5.25 ± 0.51**	7.89 ± 0.63*

Data are the mean ± SD of at least three independent experiments. *P < 0.05; **P < 0.01.*P < 0.05 versus the control, the difference was significant. **P < 0.01 versus the control, the difference was markedly significant.

**Table 4:** Depolarization of mitochondrial membrane potential of the BEL-7402 cells treated with complexes **1** and **2 **for 48 h.

Concentration (*μ*M)	Complex **1**		Complex **2**	
	Mean value of green fluorescence intensity ($\overset{-}{X}$ ± SD)	% of control	Mean value of green fluorescence intensity ($\overset{-}{X}$ ± SD)	% of control
Control	59.84 ± 0.21	100.00 ± 0.35	16.01 ± 0.09	99.99 ± 0.58
60	44.86 ± 3.99	74.96 ± 6.67	19.33 ± 2.66	120.78 ± 16.60
90	76.99 ± 3.06**	128.65 ± 5.11**	38.85 ± 1.35**	242.73 ± 8.41**
135	85.02 ± 1.37**	142.07 ± 2.28**	58.23 ± 0.50**	363.78 ± 3.13**

Data are the mean ± SD of at least three independent experiments. *P < 0.05; **P < 0.01. *P < 0.05 versus the control, the difference was significant. **P < 0.01 versus the control, the difference was markedly significant.

## Abbreviations

MTT: 3-(4, 5-Dimethylthiazoyl-2-yl) 2, 5-diphenyltetrazoliumbromide
IC_50_: 50% inhibition concentrations
Matsc: Methoxylsalicylaldehyde thiosemicarbazone
Phen: 1, 10-Phenanthroline
4-chlorosatsc: 4-Chlorosalicylaldehyde thiosemicarbazone.
